# Meta simulation approach for evaluating machine learning method selection in data limited settings

**DOI:** 10.1038/s41598-025-24627-y

**Published:** 2025-11-19

**Authors:** Mostafa Alwash, Ghadi S. Al Hajj, Ivar Grytten, Geir Kjetil Sandve

**Affiliations:** Department of Informatics, Faculty of Mathematics and Natural Sciences, University of Oslo, Oslo, Norway

**Keywords:** Computer science, Software, Statistics

## Abstract

Selecting appropriate machine learning (ML) methods for domain-specific tasks remains a persistent challenge, particularly in medicine where datasets are often small, heterogeneous, and incomplete. Traditional benchmarking strategies rely on limited observational samples, which may not capture the complexity of the underlying data-generating process (DGP). As a result, methods that perform well on available data may generalise poorly in real-world practice. We present SimCalibration, a meta-simulation framework that leverages structural learners (SLs) to infer an approximated data-generating process from limited data and generate synthetic datasets for large-scale benchmarking. This framework enables systematic evaluation of machine learning method selection strategies in settings where the true data-generating process is either known or can be approximated, allowing both validation against the ground truth and the generation of synthetic observations inferred from sparse samples. In rare disease research for example, where patient cohorts are inherently small, causal relationships are often conceptualised as directed acyclic graphs (DAGs). In this work, such structures are approximated directly from observational data, extending the utility of small datasets by enabling investigators to benchmark ML methods in a controlled simulation setting before deploying them in practice. This reduces the risk of selecting models that generalise poorly and supports more reliable decision-making in sensitive healthcare contexts. Experiments demonstrate that (a) structural learners vary in their ability to recover representative simulations for benchmarking, (b) structural learner-based benchmarking reduces variance in performance estimates compared to traditional validation, and (c) in some cases, structural learner-based approaches yield rankings that more closely match true relative performance than those derived from limited datasets. These findings highlight the value of simulation-based benchmarking for domains where drawing generalisable conclusions is critical, such as medicine, and offer greater transparency into the assumptions underlying predictive decisions.

## Introduction

Selecting the most suitable ML method for a given task is fundamentally a problem of *benchmarking*: evaluating candidate methods on available data to guide model choice. In practice, however, benchmarking is often unreliable in domains where practitioners have limited access to the true DGP, often exemplified by a single dataset. This challenge is particularly evident in medicine—for example, in clinical research, barriers such as ethics and logistics constrain data collection and result in narrow observational cohorts. Standard validation procedures, such as hyperparameter selection or splitting a dataset into training, validation, and test sets^1,2^, assume that the observed data are representative of the true DGP. In data-limited settings, this assumption rarely holds, leading to performance estimates that are error-prone and potentially misleading.

One approach to address this challenge has been the use of simulations, where researchers construct synthetic datasets intended to reflect real-world complexity. In domains where large observational datasets are available—such as the UK Biobank—practitioners often benchmark methods directly on these resources. However, in settings where such data are scarce, simulations provide an alternative means of evaluation. In epidemiology, for example, simulations are frequently defined by manually specifying DGPs using domain expertise and causal assumptions^3,4^. While valuable, the realism of these simulations depends heavily on the accuracy of the assumed structures and parameter choices. As a result, they are difficult to scale and provide no systematic way to assess how well they approximate the underlying processes they aim to model.

SLs provide a data-driven mechanism to infer DGPs directly from empirical observations. These methods estimate DAGs that encode probabilistic relationships among variables, offering a principled way to approximate underlying structures even from limited data. Leveraging SLs to orchestrate simulation-based learning allows investigators to generate large numbers of controlled synthetic datasets that explore plausible variations of the data while maintaining a formal connection to the observed samples, thereby enabling more robust benchmarking of ML method selection.

In this study, we introduce SimCalibration, a meta-simulation framework designed to evaluate ML method selection strategies under conditions where the true DGP is known. The framework allows comparison between conventional validation-based benchmarking and simulation-enhanced strategies that use SLs to infer DGPs. By situating benchmarking within a meta-simulation—where investigators have access to both limited samples and the ground-truth DGP—we are able to systematically test how well different strategies approximate true model performance.

This work makes three contributions. First, it defines a formal meta-simulation setting for evaluating ML benchmarking strategies in data-limited domains. Second, it introduces the SimCalibration package, which operationalises this evaluation through an open-source, extensible framework. Third, it demonstrates empirically that SL-based benchmarking can reduce variance in performance estimates and, in some cases, more accurately recover the true ranking of ML methods than conventional validation alone.

## Related work

The following reviews key literature in simulation, Bayesian network structure learning, and meta-learning, highlighting their relevance to simulation-based ML method selection.

### Machine learning and simulation

ML methods have increasingly been applied in medicine to forecast outcomes such as hospitalisation and mortality. For instance^5^, demonstrates how a single ML model can effectively predict these outcomes, showcasing the conventional reliance on historical data patterns to support clinical decision-making. While such applications highlight the promise of ML, they also reflect a traditional paradigm wherein models depend heavily on retrospective data and assume static underlying DGPs.

However, a persistent blind spot in ML applications lies in the limitations of real-world medical data. Clinical datasets are often incomplete, imbalanced, and collected under varying conditions, which challenges the robustness and generalisability of predictive models. These limitations not only hinder model training but also compromise the reliability of predictions in new or unseen medical contexts.

To mitigate these challenges, there is a growing shift toward simulation-enhanced learning. Simulation techniques now support ML through data enrichment strategies such as transfer learning, data augmentation, and bootstrapping, which help overcome data scarcity and variability by generating synthetic datasets^6^. These techniques enable models to generalise better by exposing them to diverse, controlled variations of medical phenomena which would otherwise be unobserved.

Simulation methodologies play a particularly transformative role in medicine, where ethical, practical, or logistical constraints often limit the collection of comprehensive real-world data. Through simulation, researchers can design and explore hypothetical medical scenarios, infer unobserved dynamics, and validate models against synthetic cohorts. These synthetic datasets do not merely fill data gaps—they offer structured approximations of complex patterns, disease trajectories, and treatment responses that may be rare or entirely absent in traditional datasets. The ability of simulations to replicate and manipulate DGPs within a controlled environment equips ML with more holistic exposure, enabling them to infer generalisable patterns and make more robust, real-world conclusions.

### Bayesian networks and structure learning

DAGs have emerged as a powerful modelling language for Bayesian Networks, offering a consistent and mathematically grounded way to represent structural, causal, and parametric assumptions. DAGs provide an intuitive framework for visualising relationships among variables, in which nodes represent variables and edges denote probabilistic dependencies. This makes DAGs not only analytically robust but also broadly applicable across domains where understanding and interrogating DGPs is essential.

Manually specified DAGs have proven particularly effective in simulation-based studies. For example, Setoguchi et al.^3^ used simulations in pharmacoepidemiology to assess bias and efficiency in propensity score methods under controlled conditions such as rare outcomes or moderate treatment effects. Similarly^4^, explored how different covariate selection strategies, guided by DAGs, perform in simulated case-control studies. They found that full DAG-based models typically matched or outperformed simplified alternatives, particularly when the assumed relationships between variables aligned with the true underlying DGP. These works demonstrate how manually constructed DAGs, even when imperfect, allow researchers to simulate complex scenarios and evaluate how variable relationships—such as indirect influences or hidden biases—can affect analytical outcomes in a controlled way.

While manually defined DAGs offer transparency and control, they rely on domain expertise and are limited in scalability. SLs address this by inferring DAG structures directly from empirical data^7–9^, offering a data-driven alternative for approximating underlying DGPs. These algorithms enable scalable simulation and benchmarking by producing interpretable models that capture empirically grounded dependencies. This makes SLs particularly valuable in meta-simulation contexts, where robust evaluation of ML methods depends on realistic, yet controlled representations of underlying variable relationships.

SLs have gained attention for their potential to bridge empirical data and synthetic generation by learning underlying structures directly from observations. However, ongoing challenges remain in evaluating how well the resulting simulations preserve critical characteristics of the original data. For instance, Cao et al.^10^ propose benchmarking criteria such as the retention of statistical properties, preservation of biologically relevant signals, and computational scalability. As benchmarking standards evolve, the literature increasingly highlights how SL-generated DAGs can encode latent or unmeasured relationships often absent in observational data, enabling the creation of synthetic datasets that not only mirror observed patterns but also extend beyond them. By incorporating these additional insights, SLs offer a principled way to stress-test and refine ML method selection under scenarios that approximate real-world complexity while maintaining known ground truths.

This study focuses on SLs due to their use of statistical heuristics^11^, which allow for estimation of DAGs from real-world datasets. We applied a suite of SL algorithms (hc, tabu, rsmax2, mmhc, h2pc, gs, pc.stable) using default parameters from the *bnlearn* library, including constraint-based, score-based, and hybrid approaches. Each category offers distinct assumptions and trade-offs:

- Constraint-based methods: identify edges via conditional independence testing. They are computationally efficient but sensitive to statistical thresholds.
- Score-based methods: evaluate candidate DAGs by optimising a scoring function. While more flexible, they are computationally intensive and prone to overfitting without regularisation.
- Hybrid methods: integrate both strategies, first reducing the search space through constraints, then optimising DAG selection within this subset.

### Meta-learning, hyperparameterisation, and Prior-data fitted networks

Recent advances in machine learning theory have emphasised strategies that operate across tasks rather than within a single dataset, a perspective formalised in meta-learning. By training models over distributions of tasks, meta-learning approaches aim to enable few-shot generalization, where performance transfers to novel datasets with minimal additional supervision. This orientation contrasts with conventional model training, which presupposes fixed inductive biases and dataset-specific optimisation.

A related strand of research explores how hyperparameterisation and AutoML frameworks can be extended beyond dataset-level tuning to capture *priors over methods themselves*. Rather than selecting models de novo for each dataset, AutoML systems increasingly rely on performance priors learned from multiple tasks, effectively transforming model selection and hyperparameter choice into meta-level inference problems.

These developments converge in the emergence of foundational models for tabular learning, most prominently by Prior-data Fitted Networks (PFNs). Models such as TabPFN^12^ pretrain a transformer-based predictor on millions of synthetic datasets, many generated from structural causal models and DAGs. By learning from this synthetic distribution, TabPFN approximates Bayesian predictors and achieves zero-shot classification and regression on unseen datasets, with superior performance compared to tuned ensembles. Rather than relying on hand-crafted inductive biases, PFNs employ in-context learning to recover diverse algorithms directly from examples of input–output behavior, shifting the design burden from algorithm specification to dataset generation. This underscores a broader trend: synthetic data, when grounded in causal structure, can serve not only as a tool for pretraining predictive models but also as a foundation for evaluating and comparing competing methods—a principle that informs the simulation-based benchmarking approach developed in this study.

## Materials and methods

This section outlines the methodology and materials used in the meta-simulation. It details the methodological approach, toolsets and configurations used for data generation, structure learning, and machine learning.

### Methodology overview

The meta-simulation workflow adopts a practitioner’s perspective: whereby an investigator has access to a limited dataset sampled from an underlying DGP. The objective is to assess whether simulation-based strategies improve method selection compared to using the limited dataset directly.

**Fig. 1:**
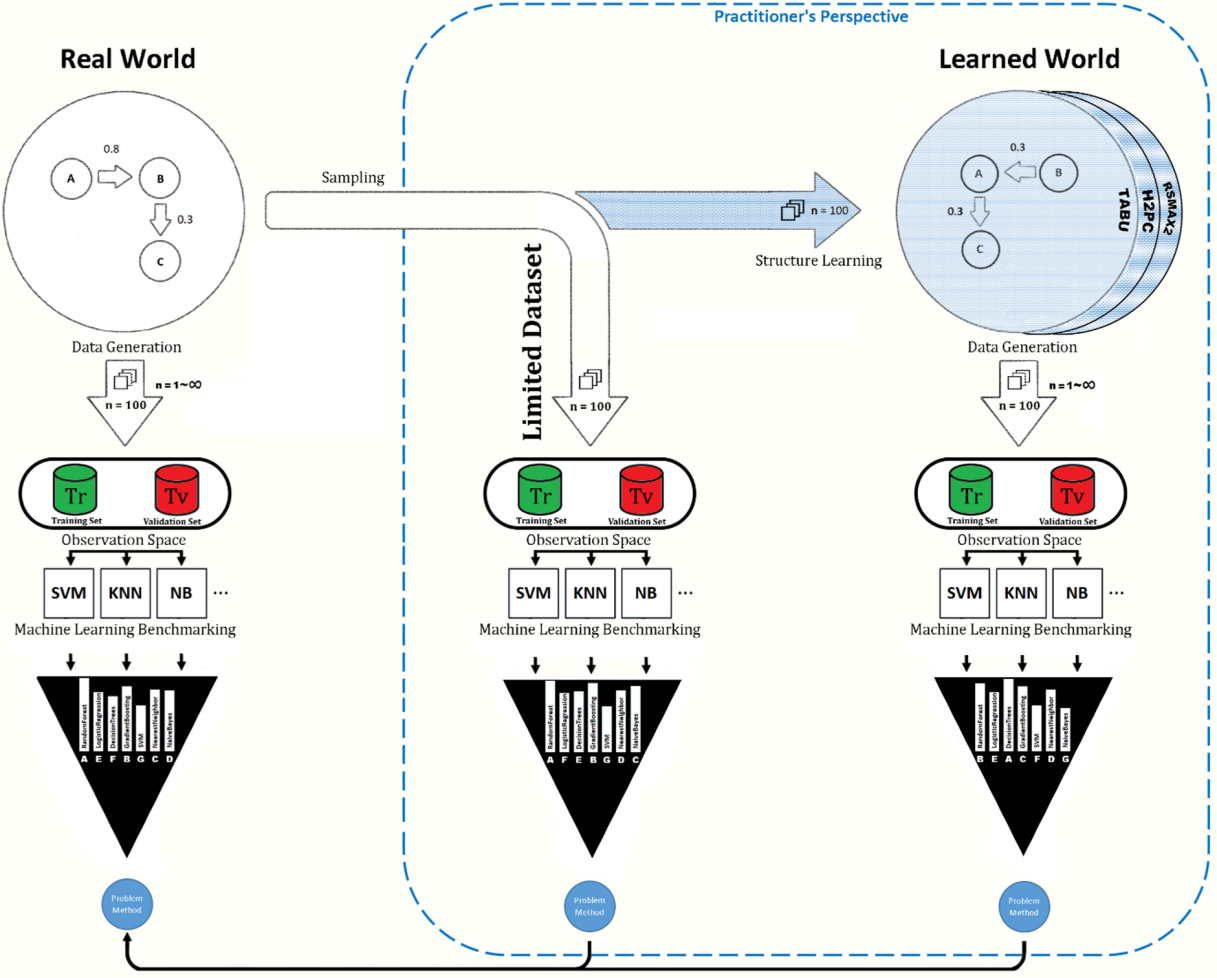
Meta-Simulation Methodology Overview.

Figure 1 summarises the meta-simulation workflow. From a known DGP (top-left), the practitioner accesses a limited dataset. Two strategies are explored:

1. Direct benchmarking using the sampled dataset (bottom-middle).
2. Apply simulation-based benchmarking using SLs (top-right) to estimate the DGP and draw synthetic samples to benchmark methods (bottom-right).

The meta-simulation investigator, with full access to the true DGP, generates multiple datasets to estimate asymptotic ML performance (bottom-left). These serve as a reference to evaluate practitioner strategy performance.

The meta-simulation is configured by defining the following parameters. The specific values reported here correspond to those used to generate the results presented in this study:

- Ground-truth DGP (GT-DGP): WIN95PTS, ANDES.
- SL algorithms: hc, tabu, rsmax2, mmhc, h2pc, gs, pc.stable.
- ML methods: RandomForestClassifier, DecisionTreeClassifier, MLPClassifier, AdaBoostClassifier.
- Dataset sizes: N_train = 200, N_test = 200.
- Repetitions: N_practitioner = 10, N_SL = 500, N_truth = 1000.
- Cross-validation: K-folds = 1 (no additional folds).

### The approach is reflected in the following pseudo code


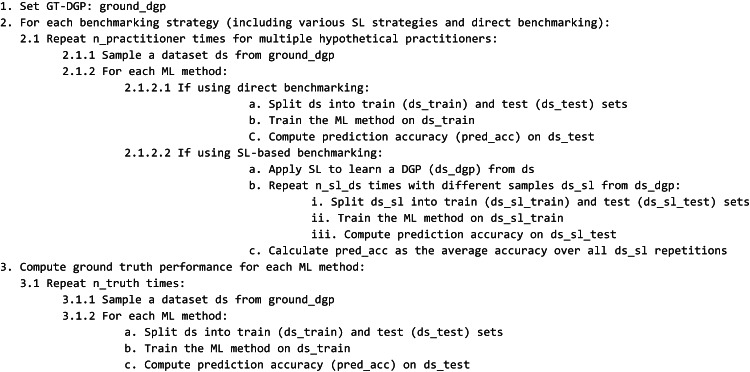


The computed results from the pseudocode above is output as generated tables and figures, comparing estimated ML performance across practitioner strategies with the asymptotic ground-truth performance of the ML methods.

### Data generation

**Fig. 2:**
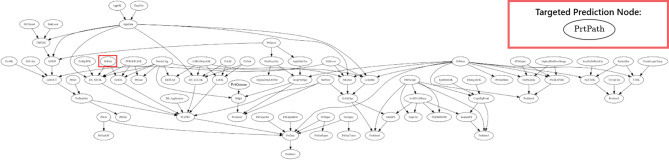
WIN95PTS DAG (target node: PrtPath).

**Fig. 3:**
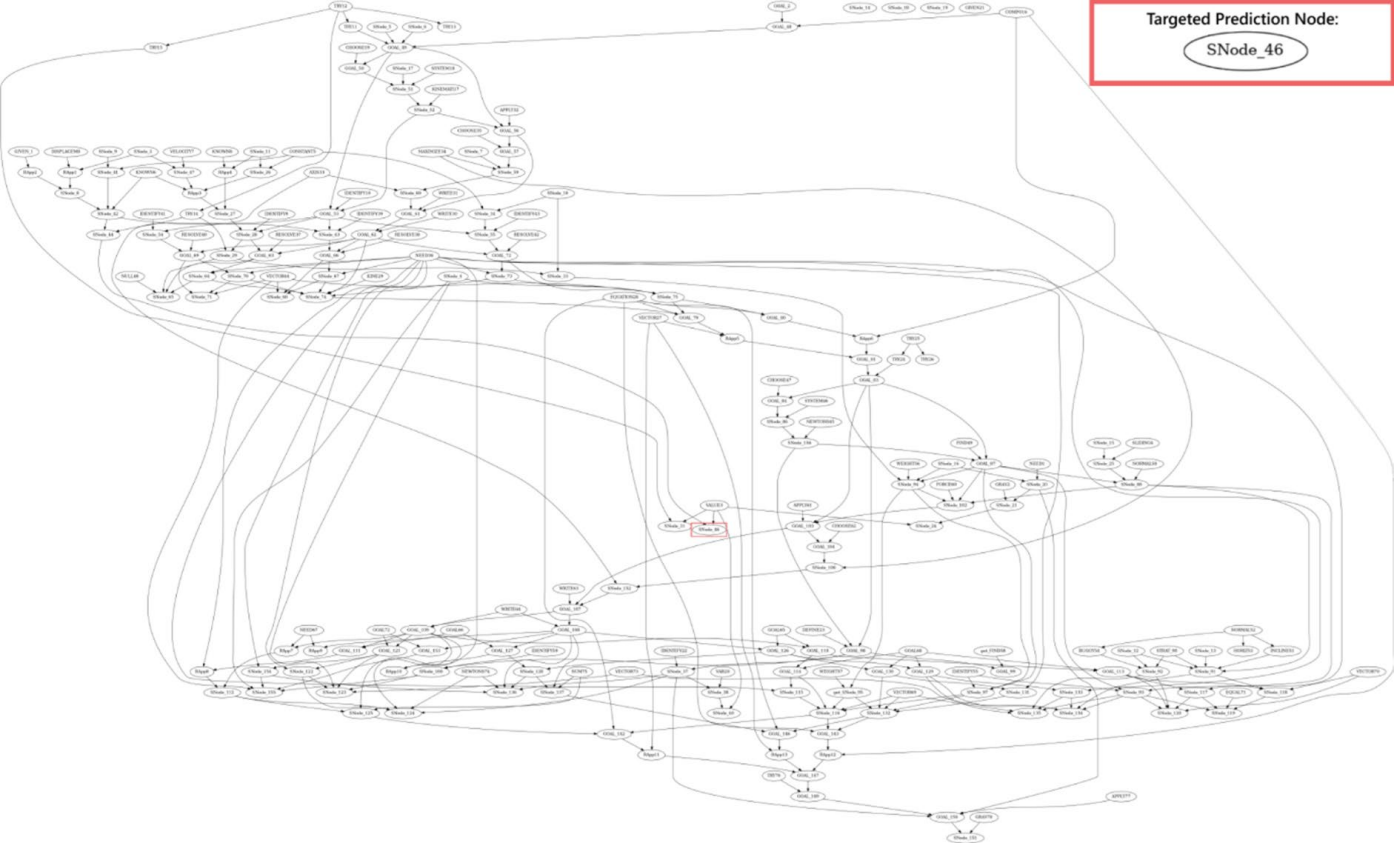
ANDES DAG (target node: SNode_46).

Bayesian DAGs (WIN95PTS, ANDES) were sourced from the publicly available Bnlearn Bayesian network repository^13^ and used as GT-DGPs. This repository was chosen due to availability of large network sizes, that represent complex processes, and make available conditional probability tables (i.e., model parameters) which are not consistently reported in the literature^7^. The adopted DAGs had been widely used in benchmarking studies^14–16^ which made them relevant for the purposes of this study. Each DAG was applied to a classification task, with the target variable chosen based on its ability to generate variance among ML methods. All nodes were represented as independent features, and a selected node (outlined in a red border) represented the predicted target variable in the DAGs, as depicted in Figs. 2 and 3. For the WIN95PTS network, the node selected for prediction was where the Markov boundary (minimal Markov blanket) consisted of two child nodes, two spouse nodes (parents of its children) and no parents. This leads to a challenging prediction problem. In contrast, the ANDES network’s target node, with a simpler Markov boundary contained only two parent nodes, representing a less complex prediction task. In both cases, the data-generation process includes many variables that are independent of the node to be predicted, thus adding non-informative elements to the prediction task.

To operationalise these DAGs, the data simulation framework DagSim^17^ was employed, providing a high-level modular tool for encoding ground-truth models. DagSim enables users to design simulation models by specifying variables (nodes) and dependencies (edges) using Python/YAML, generating data samples through forward sampling. This functionality makes DagSim ideal for generating dimensional simulation backbones (i.e., true DAGs) that served as ground-truth models for the basis of (Pretended) Real-Worlds (PRWs) in the meta-simulation.

### Data processing

To perform and evaluate ML performance, we used scikit-learn (https://scikit-learn.org*)*, a widely adopted, well-documented, and actively maintained ML package for Python. Scikit-learn provides a comprehensive suite of tools for data manipulation, preprocessing, and model evaluation, as well as standardised implementations of ML algorithms for predicting an unknown $\:f\left(x\right)$ given data observations (i.e., features and labels). Standardised implementations of classification algorithms were selected because they allow for consistent configuration and parametric calibration in this experimental context. Four supervised learning estimators were employed to represent a diverse set of methods: *RandomForestClassifier*, *MLPClassifier*, *AdaBoostClassifier*, and *DecisionTreeClassifier*. Hyperparameter tuning was incorporated into the meta-simulation. For the *DecisionTreeClassifier*, we evaluated two settings of the criterion parameter: gini (Gini impurity) and entropy (information gain). This illustrates how parameter variation can be incorporated into the benchmarking process, with these variants treated as distinct candidate methods. All experiments were executed using Python 3.8 with the SimCalibration package on a system running Ubuntu 20.04 with an Intel i7 processor and 32 GB RAM. Random seeds in Python’s random and scikit-learn libraries were fixed at 42. Execution times were approximately 14 h for WIN95PTS and 30 h for ANDES on the specified hardware. To replicate the results, investigators can input the same parameter configurations listed previously to the SimCalibration package; this will reproduce the same outcomes reported in the manuscript.

### Evaluation notation

To evaluate how closely practitioner strategies approximate the ground truth, three perspectives were assessed. For a meta-simulation, let $\:=\{{M}_{1},\dots\:,{M}_{K}\}$ denote the set of ML methods, and $\:Acc(M,D)$ the accuracy of method $\:M$ trained and tested on dataset $\:D$.

1. Ground Truth Performance.

- For each method $\:M$, the asymptotic ground truth performance is estimated from $\:{N}_{truth}$ repetitions:

$$\:{\mu\:}_{M}^{true}=\frac{1}{{N}_{truth}}\sum\:_{i=1}^{{N}_{truth}}Acc(M,{D}_{GT}^{\left(i\right)})$$

2. Practitioner Estimate.

- Based on limited datasets drawn from the ground-truth DGP:

$$\:{\mu\:}_{M}^{prac}=\frac{1}{{N}_{prac}}\sum\:_{i=1}^{{N}_{prac}}Acc(M,{D}_{GT,small}^{\left(i\right)})$$

3. SL-based Estimate.

- For a given structural learner $\:L$, each limited dataset is used to infer a DGP from which $\:{N}_{SL}$ datasets are sampled:

$$\:{\mu\:}_{M}^{L}=\frac{1}{{N}_{prac}}\sum\:_{i=1}^{{N}_{prac}}\left(\frac{1}{{N}_{SL}}\sum\:_{j=1}^{{N}_{SL}}Acc(M,{D}_{L}^{(i,j)})\right)$$

From these, three evaluation perspectives were derived:

- True Difference Estimation:

Bias in absolute performance is measured by:

$$\:{\varDelta\:}_{M}^{L}=\:{\mu\:}_{M}^{L}-{\mu\:}_{M}^{true}$$

- Relative ML Difference Estimation:

For method selection, relative performance is more relevant than absolute accuracy. We define the centred performance as:

$$\:{\stackrel{\sim}{\mu\:}}_{M}^{X}={\mu\:}_{M}^{X}-\frac{1}{K}\sum\:_{k=1}^{K}{\mu\:}_{{M}_{k}}^{X},\:X\in\:\left\{true,prac,L\right\},$$

Comparing $$\:{\stackrel{\sim}{\mu\:}}_{M}^{L}$$ against $$\:{\stackrel{\sim}{\mu\:}}_{M}^{true}.$$.

- Ranking Consistency:

The rank of each method under strategy $\:X$ is given by:

$$\:{r}^{X}\left(M\right)=rank\left({\mu\:}_{M}^{X}\right),\:X\in\:\left\{true,prac,L\right\}.$$

Alignment with the ground truth ranking is assessed by comparing $\:{r}^{L}\left(M\right)$ and $\:{r}^{true}\left(M\right)$.

## Results

### DAG and distribution fidelity of SL learners

**Fig. 4:**
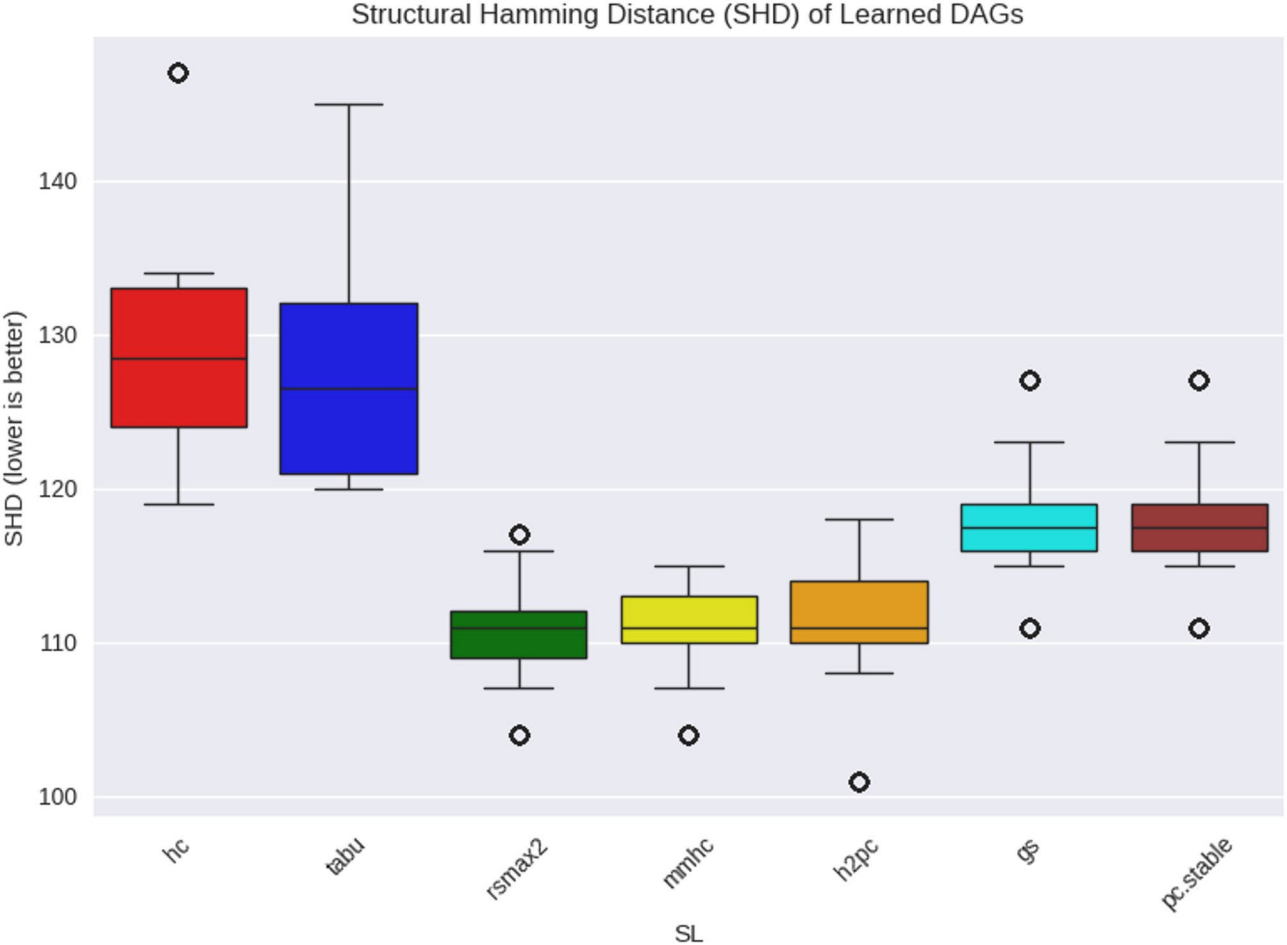
WIN95PTS DAG Fidelity comparison in Learned DAGs to true.

**Fig. 5:**
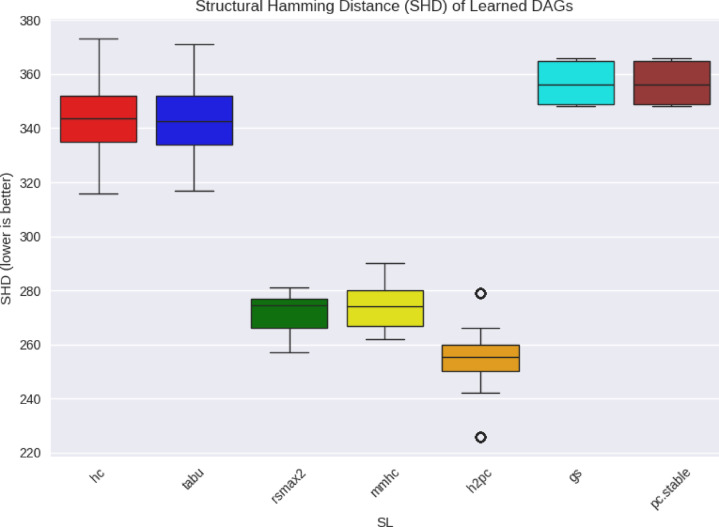
ANDES DAG Fidelity comparison in Learned DAGs to true.

We first assessed how closely the SL-inferred DAGs approximated the ground-truth DAGs by computing the Structural Hamming Distance (SHD) between adjacency matrices. Figures 4 and 5 display the SHD distributions for WIN95PTS and ANDES, respectively.

For WIN95PTS, hybrid learners such as rsmax2, mmhc, and h2pc achieved lower SHD values (median ≈ 111) than score-based learners hc and tabu (medians ≈ 126–128). Constraint-based learners (gs, pc.stable) were intermediate (≈ 117–118). In the ANDES network, hybrids again showed substantially lower SHD (medians ≈ 255–273) relative to score-based approaches (medians ≈ 341), while constraint-based approaches were least faithful structurally (≈ 355).

Notably, this structural fidelity pattern diverged from benchmarking fidelity: although hybrids were structurally closer to the true DAGs (with lower SHD), score-based learners yielded lower bias in estimated performance ($\:{\varDelta\:}_{M}^{L}$) and more consistent rankings $\:({r}^{L}\left(M\right)$). These results suggest, that for SHD, high structural fidelity is not a sufficient condition for effective benchmarking. Instead, the heuristics embedded in score-based SL algorithms may yield DAGs that deviate structurally but still preserve the relative differences in ML method performance.

**Fig. 6:**
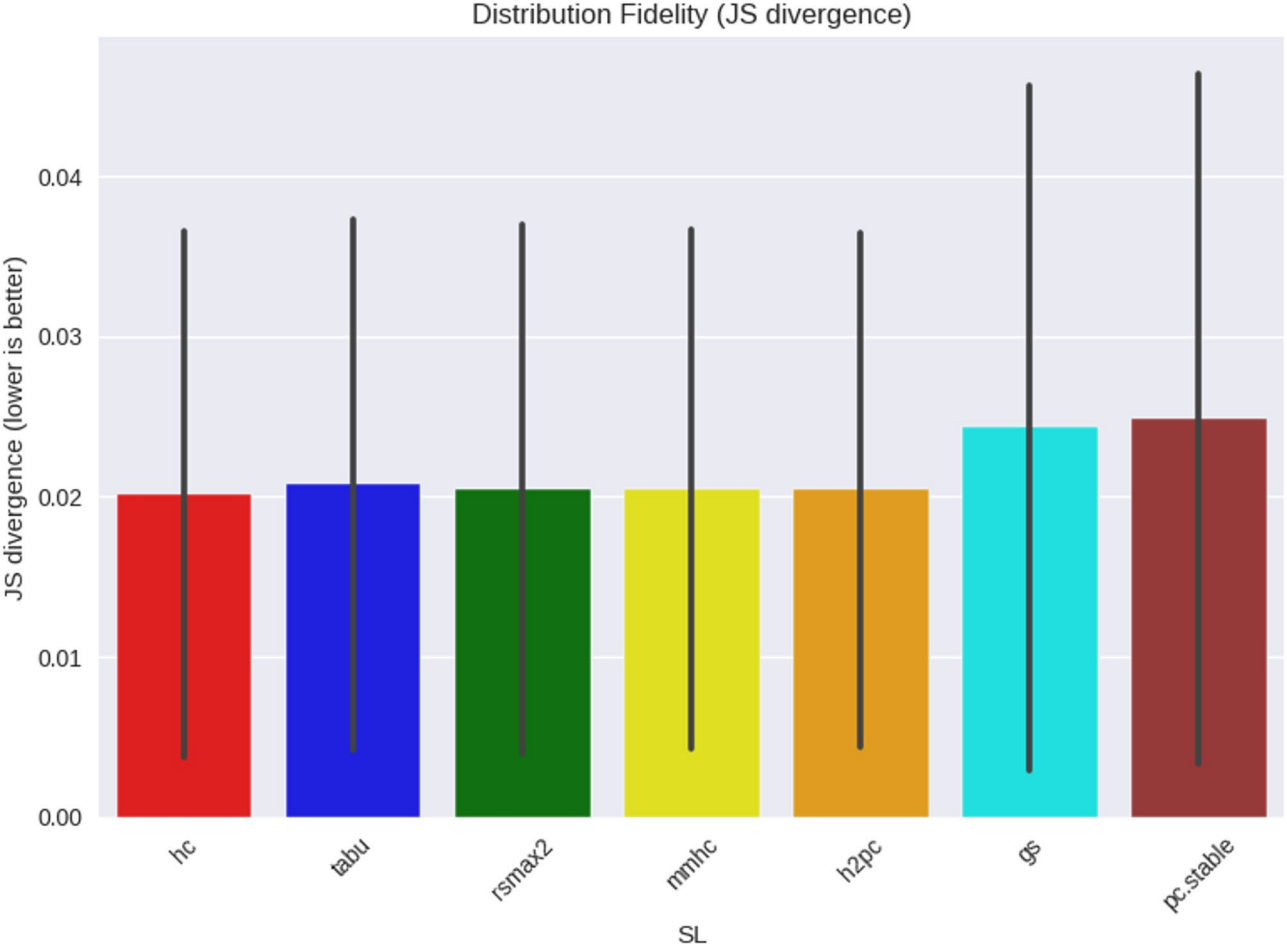
WIN95PTS DAG fidelity comparison in learned DAGs to true.

**Fig. 7:**
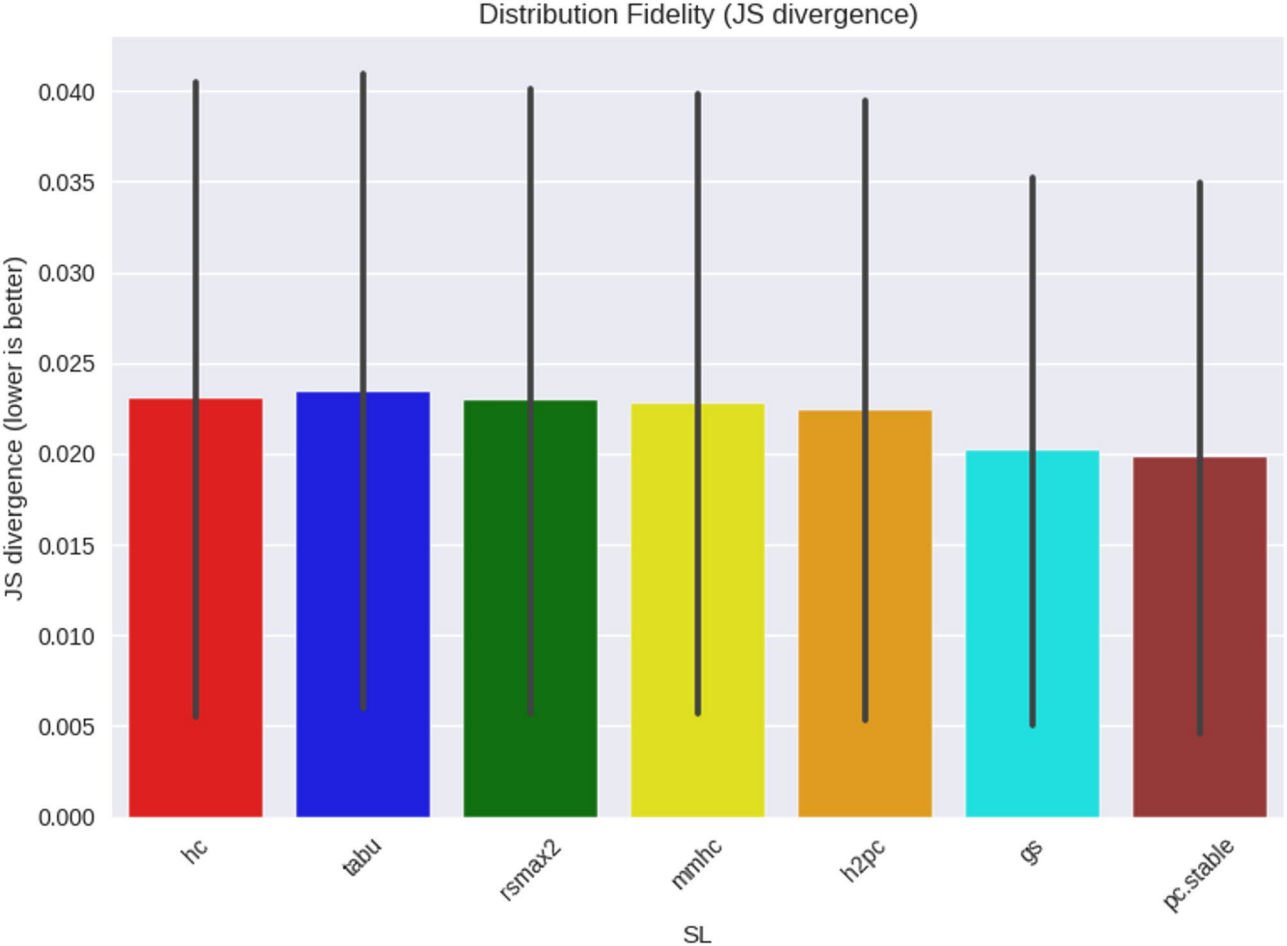
ANDES DAG fidelity comparison in learned DAGs to true.

We also compared real and synthetic data distributions using Jensen–Shannon (JS) divergence (Figs. 6 and 7). Across both networks and all learners, divergences were uniformly low (≈ 0.00–0.02, variance up to ≈ 0.04). This indicates that distributional fidelity was consistently high and unlikely to account for differences in benchmarking effectiveness.

### SL-based true difference estimation

This analysis evaluated how well SL-inferred DGPs, derived from limited data, approximate the true DGP for benchmarking ML methods. Unlike the traditional strategy (direct ML estimation from the limited dataset), SL-based strategies enable data generation, potentially reducing performance estimate variance.

**Fig. 8:**
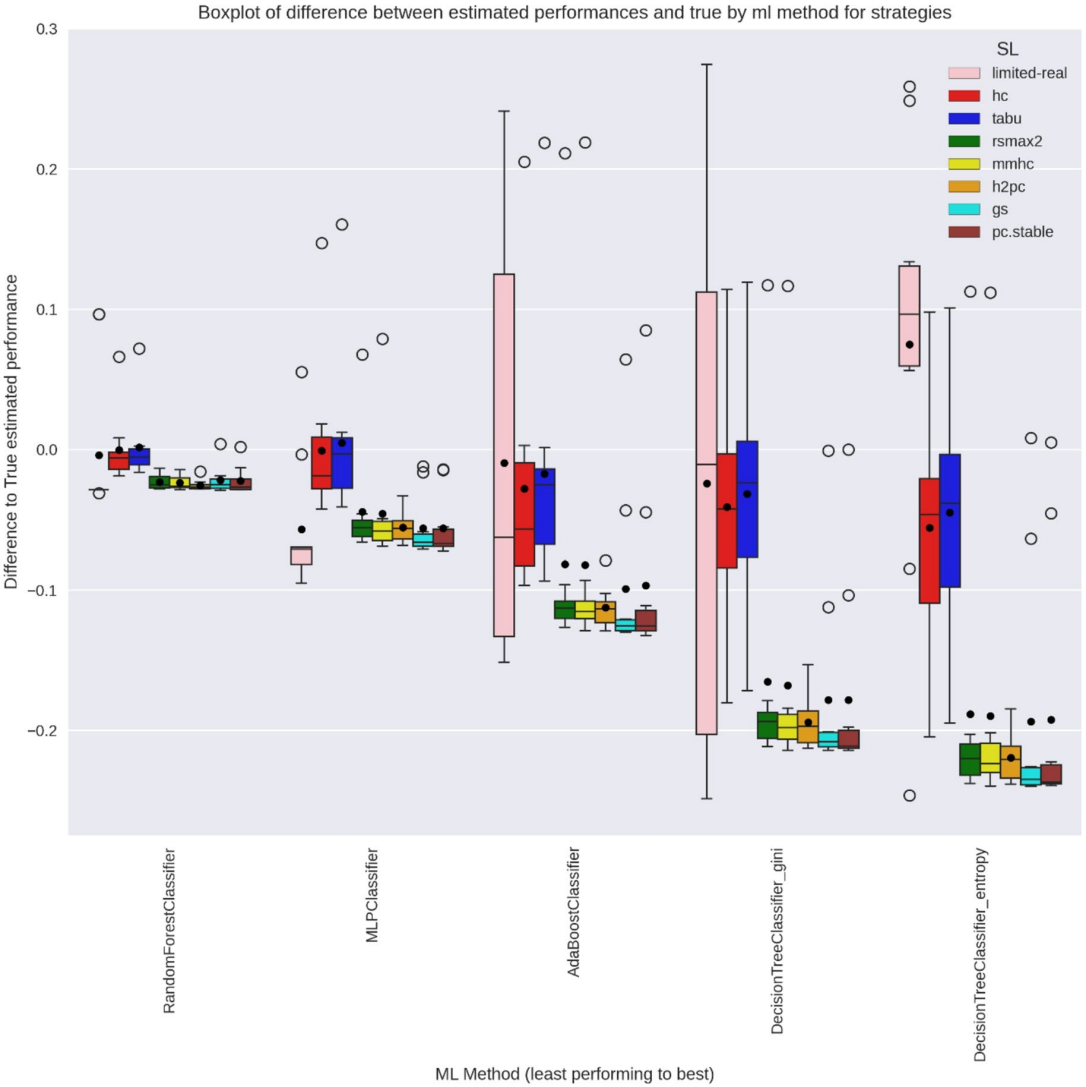
WIN95PTS Boxplot of relative difference between Y-estimates across strategies and the true real-world performance for all ML methods.

**Fig. 9:**
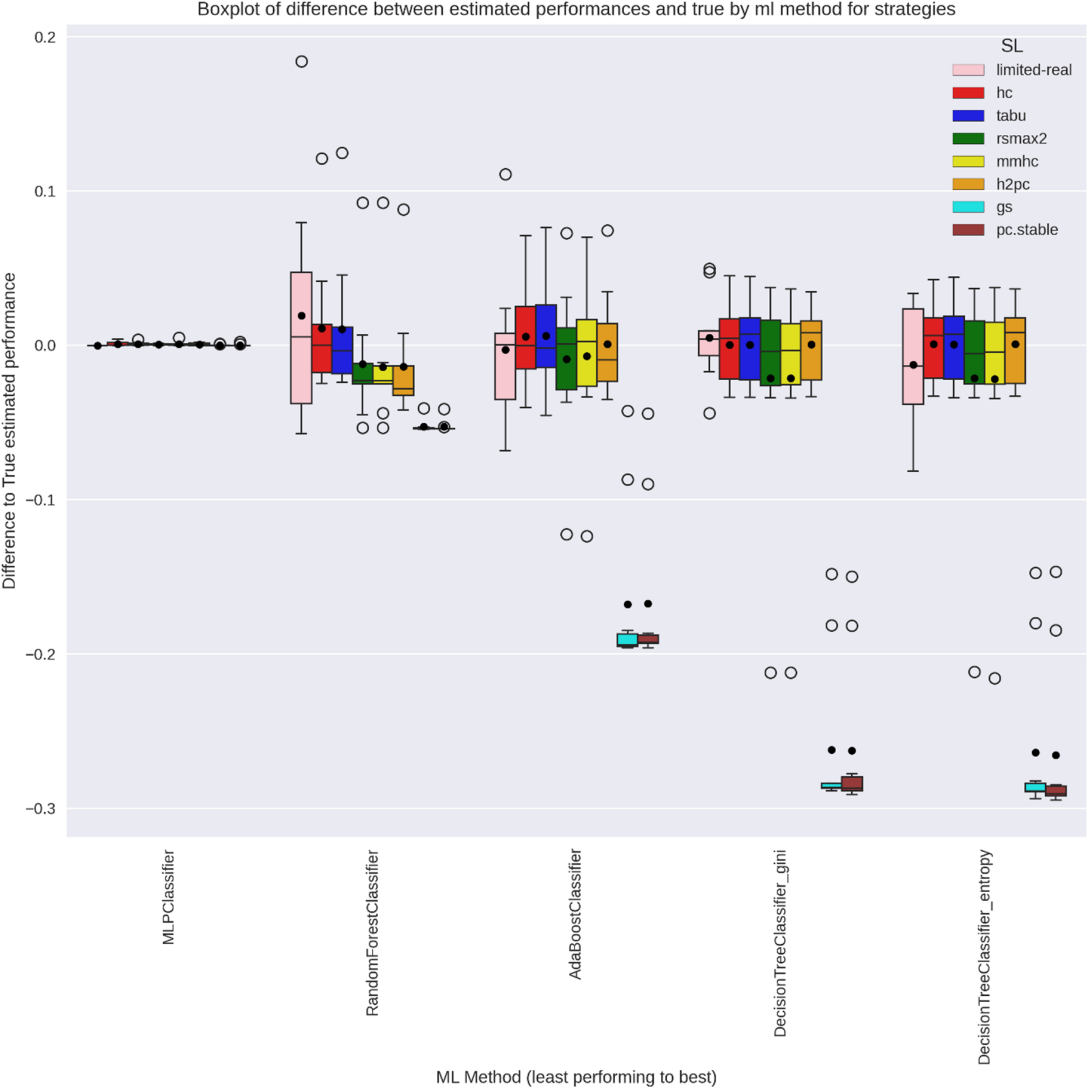
ANDES Boxplot of relative difference between Y-estimates across strategies and the true real-world performance for all ML methods.

Figures 8 and 9 display the relative difference between estimated and true ML performance across strategies. Since the limited-real strategy samples directly from the true DGP, the ML performance estimates are intrinsically unbiased. However, the limited size of the samples leads to a high inter-practitioner variance. SL-based approaches may introduce bias ($\:{\varDelta\:}_{M}^{L}$) in estimated ML performance $\:({\mu\:}_{M}^{L}$) when the inferred DGP deviates from the true distribution $\:\left({\mu\:}_{M}^{true}\right)$. Notably, the score-based SL learners (hc and tabu) introduced minimal bias in both networks, while the hybrid SL learners (all others) introduced a substantial bias, especially for the WIN95PTS network. Despite potential biases, the inter-practitioner variance is generally lower for SL learners as compared to the limited-real strategy. This results in a single practitioner being generally closer to the true ML performance when using a score-based SL strategy as compared to the limited-real.

### SL-based relative ML difference estimation

**Fig. 10:**
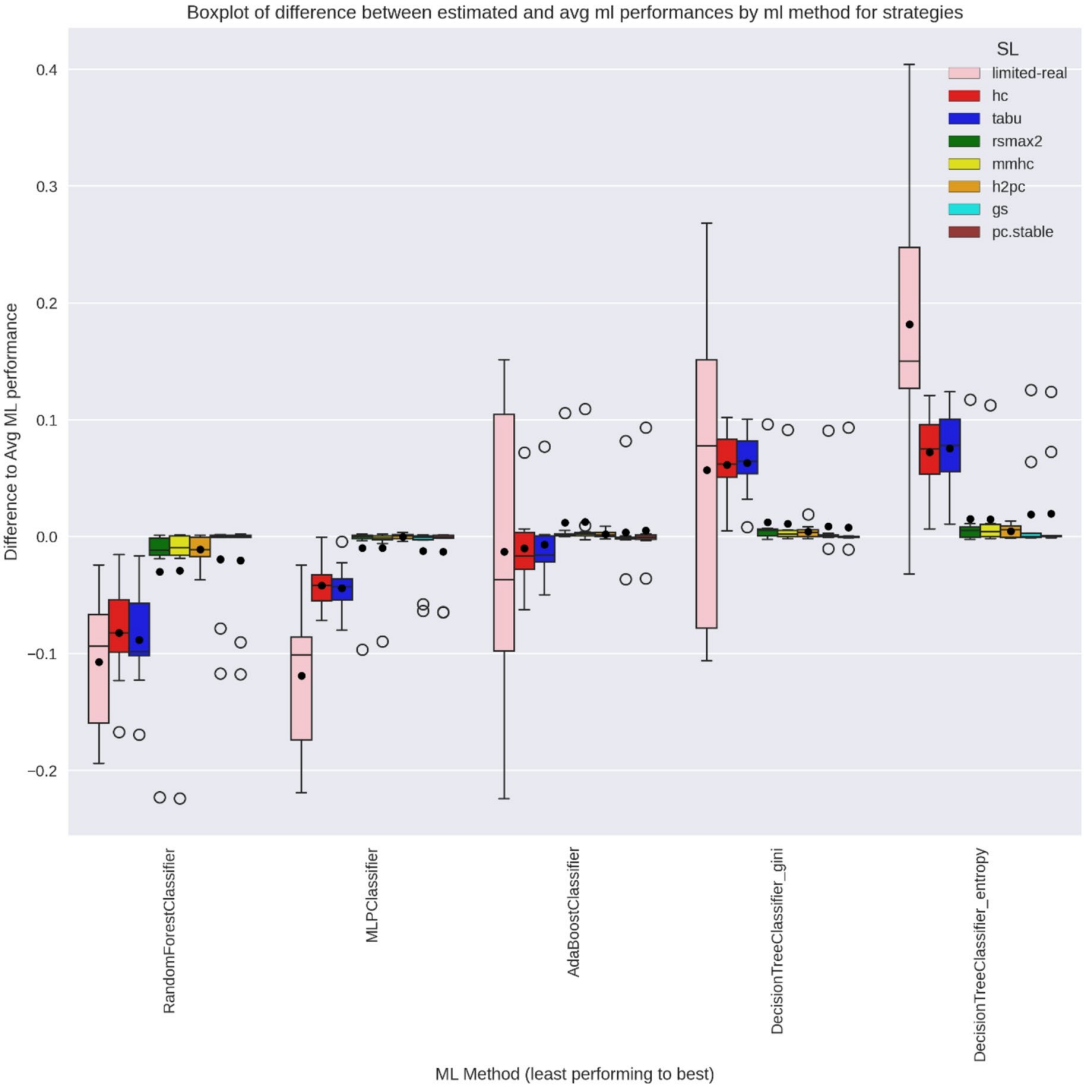
WIN95PTS Boxplot of relative difference between Y-estimates across strategies and average ML performance per practitioner repetition for all ML methods.

**Fig. 11:**
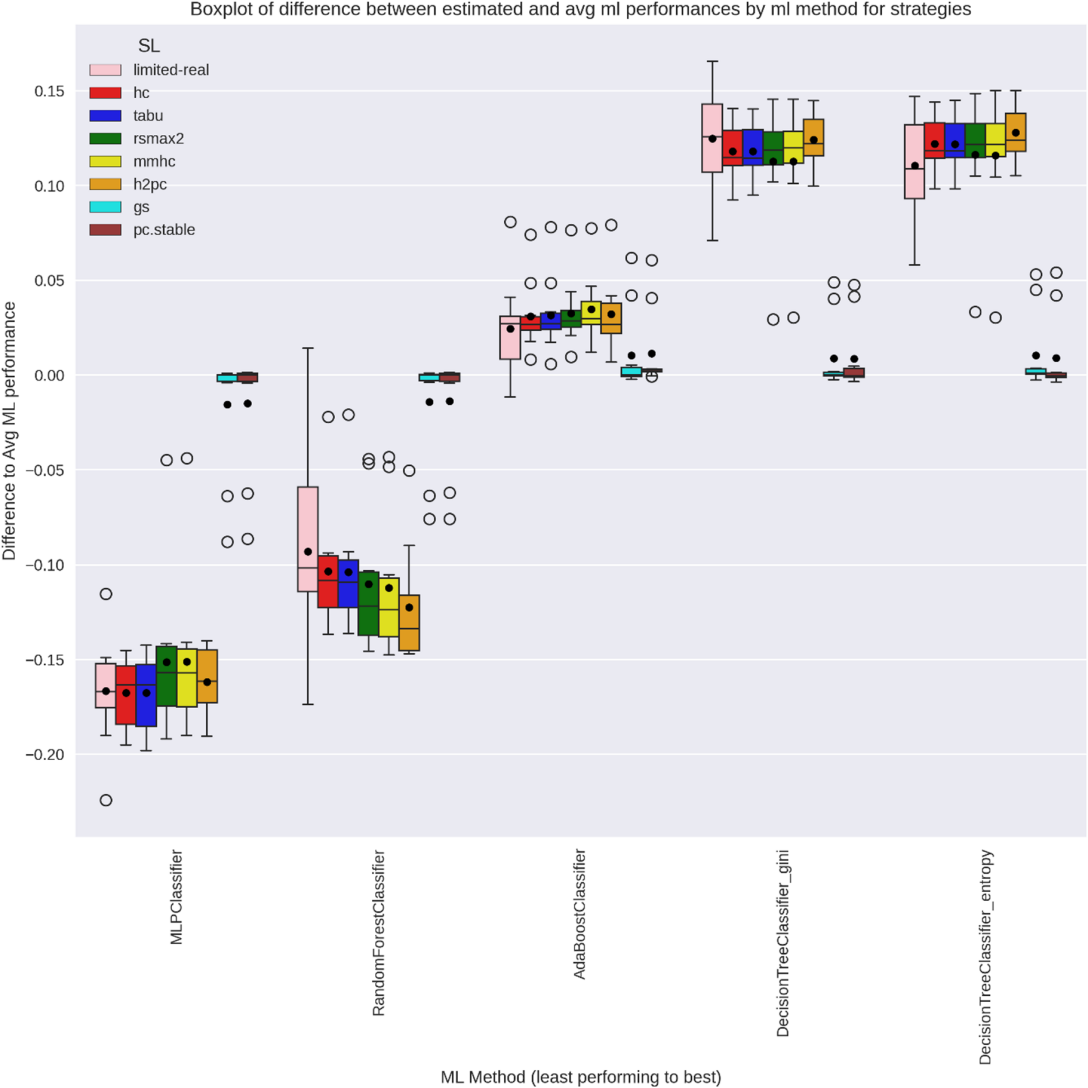
ANDES Boxplot of relative difference between Y-estimates across strategies and average ML performance per practitioner repetition for all ML methods.

For method selection, relative rather than absolute performance matters—captured here by centred relative estimates $\:({\stackrel{\sim}{\mu\:}}_{M}^{X}$) across strategies. Figures 10 and 11 show each method’s performance relative to the average across methods, per practitioner repetition and strategy. In WIN95PTS, limited-real showed high variability and overlapping method performances, making rankings unreliable. Score-based SLs achieved lower variance and clearer between-method differences. Hybrid SLs, however, exhibited limited separation, indicating difficulty in recovering true relative method performance.

### ML method ranking consistency

**Fig. 12:**
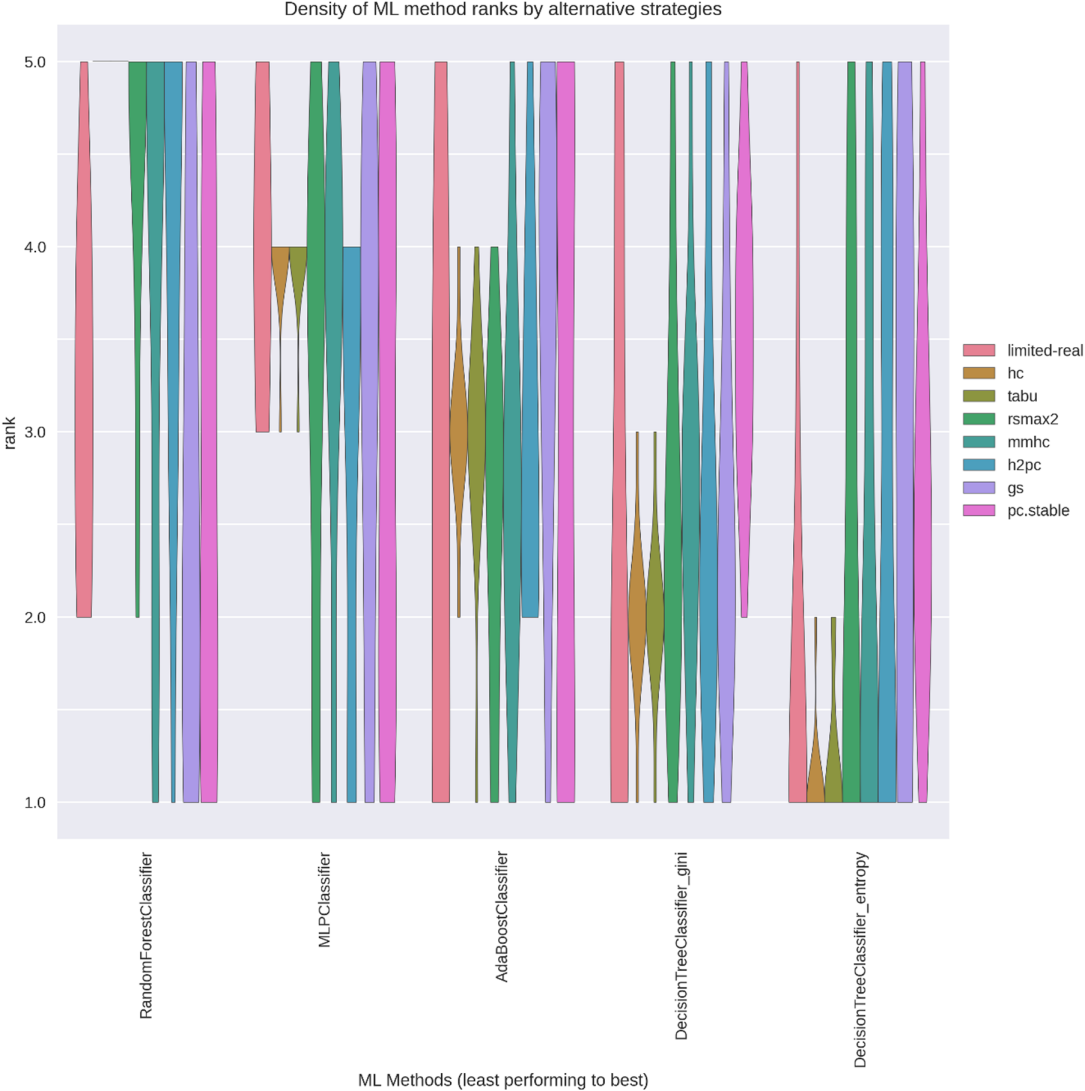
WIN95PTS violin plots of rank-order density by SL across ML methods.

**Fig. 13:**
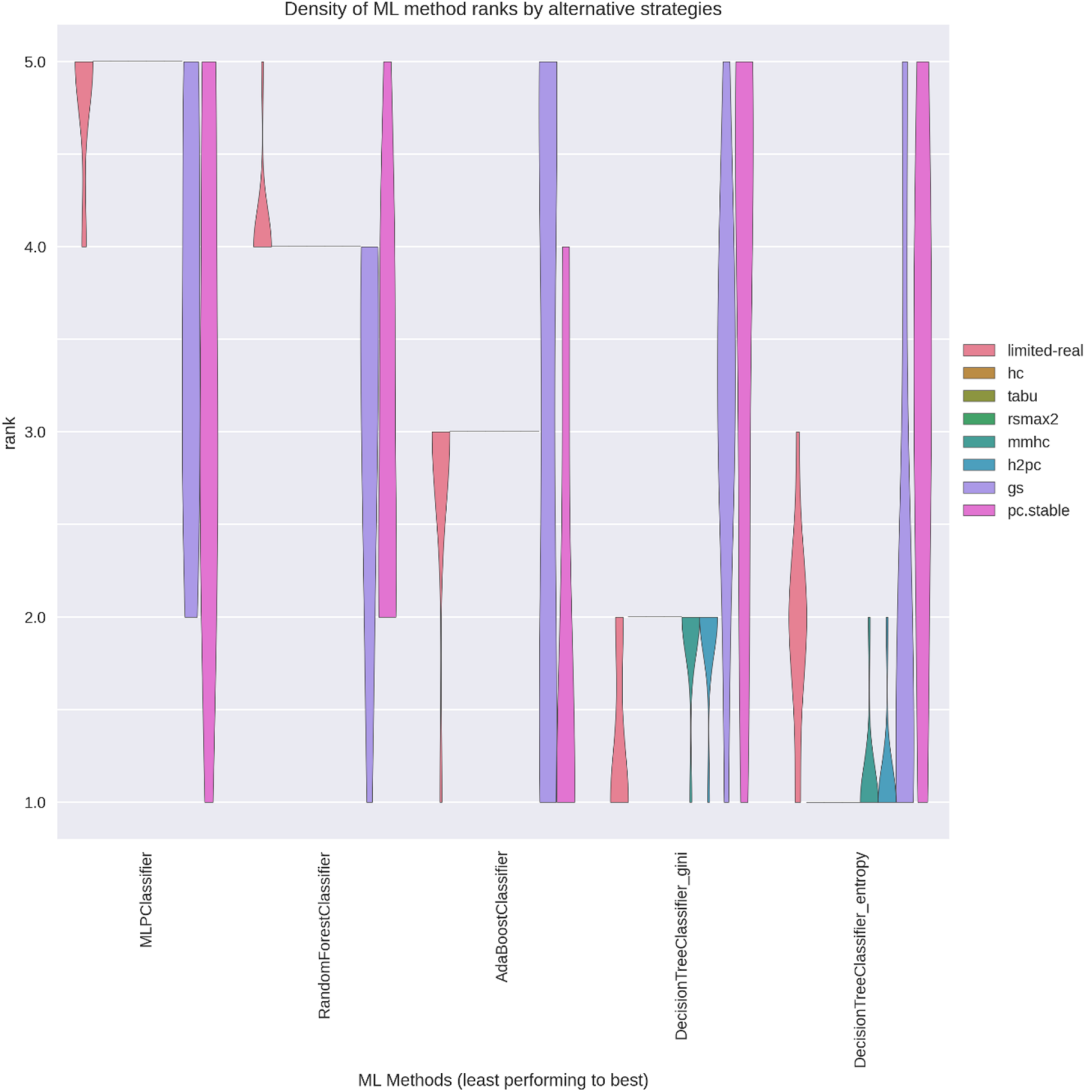
ANDES violin plots of rank-order density by SL across ML methods.

Figures 12 and 13 show the rank-order consistency of ML methods, measured by comparing inferred rankings ($\:{r}^{X}\left(M\right)$) against the true ranking $\:\left({r}^{true}\right(M\left)\right)$. Ideal rank distributions would concentrate all probability mass along the diagonal (rank 5 for the leftmost ML method and rank 1 for the rightmost ML method), indicating high alignment with the true performance order. In ANDES, all strategies—limited-real, score-based SLs, and select hybrid SLs (excluding gs and pc.stable)—closely matched true rankings. For WIN95PTS, score-based SLs outperformed others in aligning with the true rank order.

These results indicate that score-based SL strategies offered the best balance of low variance in performance estimates ($\:{\mu\:}_{M}^{L}$) and minimal bias relative to the ground truth ($\:{\varDelta\:}_{M}^{L}$), particularly for more challenging predictions like WIN95PTS. This highlights their value in contexts where limited datasets may otherwise impede robust method selection.

## Discussion

Selecting the right ML method in data-constrained environments remains a critical challenge across domains. In response, many fields have defaulted to a small set of standard methods shown to perform well or have relied on manually crafted simulations for benchmarking. This study offers an alternative: leveraging SL-inferred DGPs to simulate richer evaluation environments while remaining grounded in real data.

The central idea explored here is that while available real-world samples may be too few to fully represent the underlying system, they can still be sufficient to calibrate a DGP that preserves the relative performance of ML methods. In other words, the goal is not to reconstruct the world perfectly, but to recover enough of its structure such that benchmarking results generalise beyond the small observed dataset.

Through a meta-simulation using fully known PRWs, this work compared conventional ranking strategies on limited data against SL-based strategies that first inferred a DGP. Results showed that SL strategies varied in effectiveness. Notably, score-based SLs (e.g., hc, tabu) more reliably captured the true method ranking than the traditional limited-data approach, despite introducing bias. This suggests that the algorithmic heuristics used by SLs contribute significantly to benchmarking performance, with score-based approaches performing better than conditional independence–based approaches in the PRWs studied.

Importantly, this work also highlights limitations and trade-offs. While traditional validation is unbiased by definition, it suffers from high variance when sample sizes are small. SL-based strategies introduce bias by approximating the DGP, but this is offset by lower variance since they allow (in principle) unlimited resampling from the inferred model. This variance–bias trade-off is central to their relative effectiveness.

To further evaluate SL inference, additional analyses compared the learned DAGs with the ground-truth structures and assessed distributional fidelity of the generated data. These showed that while none of the learners fully recovered the true networks, score-based SLs tended to achieve closer structural alignment than conditional-independence or hybrid learners. At the same time, distributional differences between real and simulated data were generally small across learners. This indicates that SL-based simulations can preserve key aspects of the original DGP, even if structural recovery is incomplete.

Two limitations warrant discussion. First, the analysis was based on discrete PRWs from the *bnlearn* repository. These networks are well-established benchmarking staples in the literature and provide a valuable starting point, but they may not fully capture the diversity or complexity of real-world DGPs. In particular, the present study does not address continuous variables, confounded or misspecified DGPs, or high-dimensional, low-signal scenarios—settings that are common in real-world applications and may place different demands on both SLs and benchmarking strategies. In practice, different dynamics could emerge if custom networks were designed by domain experts who have visibility into the underlying structures and parameters of a given system, or if more challenging generative conditions were incorporated. Such extensions would further challenge the framework’s robustness and better reflect the types of problems faced in applied domains.

Second, while this study evaluated method selection, it did not address the challenges of scaling SL-based meta-simulations to accommodate a growing set of candidate methods. Computational constraints may limit the number of variants that can be thoroughly assessed, making it essential to prioritise candidates based on both practical feasibility and demonstrably high fitness. Developing strategies that guide the systematic exploration of the meta-simulation space could therefore enhance its efficiency and ensure robust evaluation across a broader set of high-quality candidates.

## Conclusion

This study demonstrates that SL-based benchmarking offers a viable alternative to conventional method selection in data-limited settings. By calibrating simulations from real data, researchers can evaluate ML methods within richer, domain-specific environments that better approximate true performance rankings.

The open-source SimCalibration framework operationalises this approach, allowing investigators to reproduce, extend, and adapt the methodology across fields where benchmarking is constrained by limited observational data. While demonstrated here on two established PRWs, the framework is general and extensible.

In summary, SL-based meta-simulation reduces variance in performance estimates, sometimes at the cost of bias, but offers a more robust and generalisable basis for ML method selection when only small datasets are available. Future research should extend this approach to more diverse DGPs, systematically explore SL tuning, and investigate applications in domains such as medicine and pharmacoepidemiology, where simulation-informed benchmarking can support both predictive modelling and causal inference.

## Data Availability

All data generated and analysed in this study are included in the article. Benchmarking results were derived entirely from synthetic data generated using the WIN95PTS (https://www.bnlearn.com/bnrepository/discrete-large.html#win95pts) and ANDES (https://www.bnlearn.com/bnrepository/discrete-verylarge.html#andes) Bayesian networks available in the bnlearn repository.These networks were imported and integrated into SimCalibration enabling full replication of methods and results. The outcomes presented in this manuscript can be reproduced by applying the same parameter setup described in the Methodology section.
